# Effect of different interfacial surface treatments on the shear bond strength of veneering ceramic and zirconia core

**DOI:** 10.1186/s12903-023-03057-0

**Published:** 2023-06-05

**Authors:** Marwa K. Youssef, Sanaa H. Abdelkader, Yasser M. Aly

**Affiliations:** Division of Fixed Prosthodontics, Conservative Dentistry Department, Faculty of Dentistry, Alexandria University, Alexandria, Egypt

**Keywords:** Zirconia, Shear strength, Veneering ceramics, Surface treatments

## Abstract

**Background:**

Several interfacial surface treatments of zirconia surfaces have been proposed to improve adhesion to ceramic veneering. However, information regarding the durability and effect of such treatments on the bond strength following such treatments is lacking.

**Aim of the study:**

This study aimed to evaluate the shear bond strength between veneering ceramic and zirconia core after different interfacial surface treatments.

**Materials and methods:**

Fifty-two discs (8 mm in diameter and 3 mm in height) were fabricated from zirconia blanks using a microtome cutting machine. Zirconia discs were divided into four groups (n = 13). Group I was subjected to air-borne abrasion using (Al_2_O_3_), group II was coated by bioglass, group III was coated with ZirLiner, and group IV was subjected to wash firing (sprinkle technique). A cylinder (4 mm in diameter and 3 mm in height) of veneering ceramic was fired on top of the zirconia core. Shear bond strength (SBS) between zirconia core and veneering ceramic was evaluated by using a universal testing machine. The data was collected and statistically analysed using One-Way ANOVA followed by multiple pairwise comparisons using Bonferroni adjusted significance level. The failure modes were assessed using a stereomicroscope for each group.

**Results:**

The highest mean bond strength was recorded in group III (17.98 ± 2.51 MPa), followed by group II (15.10 ± 4.53 MPa), then group I 14.65 ± 2.97 MPa. The lowest mean bond strength was recorded in group IV (13.28 ± 3.55 MPa).

**Conclusions:**

Surface treatments had an effect on the zirconia-veneer shear bond strength. Liner coating revealed the highest shear bond strength values, significantly higher in comparison to wash firing (sprinkle technique) .

## Background

Due to the high expectations and demands for aesthetics in routine dental treatment, the use of ceramic restorations has significantly expanded. The patient’s expectation to receive highly aesthetic restorations with good mechanical qualities has increased due to ongoing technological and procedural advancements [1].

Due to its solid polycrystalline structure, zirconia is opaque, which affects its aesthetic properties. Hence, a veneering ceramic must be used to conceal the zirconia substrate in the aesthetic zone. Zirconia-based bilayer restorations consist of an aesthetic veneering ceramic and a durable zirconia infrastructure, both of which are biocompatible [2, 3].

The bond between zirconia and the veneering ceramic depends on several factors, including the chemical bonding, mechanical clamping due to the difference in thermal expansion coefficient, wetting behaviour, and the glass transition temperature differences [4]. Thus, the zirconia surface has been altered using a variety of techniques to improve its adhesion to ceramic veneering. These techniques include roughening by air-abrasion with aluminium oxide (Al_2_O_3_), liner application, wash firing, and glass coatings [5–8].

Al_2_O_3_ particle roughening is the technique that is most frequently utilised. It enhances wettability and surface roughness while forming undercut zones, boosting the surface energy [9, 10]. Despite these positive qualities, sandblasting can damage the structure of zirconia by causing tensions and tetragonal monoclinic phase change on the zirconia surface [9, 11].

Another technique is to add a liner between the veneering ceramic and zirconia to increase the materials’ adherence by adjusting for the difference in their coefficients of thermal expansion (CTE). However, the findings were disputable. Some authors claimed that it might reduce the degree of adhesion, while others claimed that it would strengthen the binding; several claimed that it would have no effect [12–16].

Wash firing with nanofluorapatite veneering ceramic, called the sprinkle technique, was suggested by the manufacturer to enhance the in-depth shade and bond strength. In this technique, shade and glaze are mixed with the respective liquids to the desired consistency and applied to form a covering layer on the entire framework. After that, the nanofluorapatite veneering ceramic dentin powder is sprinkled on the restoration using a dry brush [17].

Bioglass coating is a technique where a glass mixture is utilised to cover the zirconia surface before applying the veneering ceramic, to increase the strength of the zirconia-ceramic interfacial bond. This glassy layer has a CTE (11.58 × 10^(−6)^K^(−1)^) comparable to that of zirconia (11.67 × 10^(−6)^ K^(−1)^) and that of fluoroapatite veneer ceramic (9.85 ± 0.25 × 10^(−6)^ K^(−1)^). This technique makes zirconia superficially more rough, consequently enhancing bonding [18, 19].

In spite of the proposal of these different interfacial surface treatments of zirconia surfaces to improve adhesion to ceramic veneering, information regarding the effect of such treatments on the bond strength is lacking. This study aimed to evaluate the effect of different interfacial surface treatments on bond strength between veneering ceramics and zirconium core. The null hypothesis was that there would be no significant difference in shear bond strength (SBS) between veneering ceramics and zirconium core after different interfacial surface treatments.

## Materials and methods

Power analysis was performed using a statistical software program (GPower 3.1.9.4; Henrich Heine University Dusseldorf) [20]. Sample size was estimated assuming 5% alpha error and 80% study power. AlOmary et al. reported a mean ± standard deviation (SD) shear bond strength of (22.02 ± 3.95) when ZirLiner coating was applied and (33.14 ± 3.82) when wash firing was applied [21]. Moezzizadeh et al. reported a mean ± SD shear bond strength of (44.84 ± 7.24) when air abrasion was performed and (38.38 ± 4.72) when bioglass coating was applied [22]. Based on comparison of means, the minimum sample size was calculated to be 12 per group, increased to 13 to make up for laboratory processing errors. The total sample size was the number of groups × number per group = 4 × 13 = 52 [20, 23].

Sample preparation and examination was done at the Conservative Dentistry Department laboratory at the Faculty of Dentistry, Alexandria University. Fifty-two discs (8 mm in diameter and 3 mm in height) fabricated from zirconia blanks were randomly divided into 4 groups: group I was the air-borne particle abrasion group, group II was the bioglass coating group, group III was the Zirliner coating, and group IV was the wash firing (sprinkle technique) group. The materials used in the current study are listed in Table 1.

**Table 1:** Materials used in the study

Material	Commercial product name	Manufacturer	Composition	Lot No.
Zirconia blanks	Sagemax NEXXZR.T	IPS e.max ZirCAD ® Ivoclar Vivadent AG, USA.	Zirconium oxide ZrO_2_≥ 89% Yttrium oxide + Y_2_O_3_ 4–6% Hafnium oxide HfO_2_≤ 5% Aluminium oxide AI_2_O_3_ < 1%	NX0884
Glass Coating	Bioglass nanoparticles	Nanostreams Egypt: NS0001	45% silica, 25% CaO, 25% Na_2_O and 5% P_2_O_5_.	
Liner coating	ZirLiner powder	Ivoclar Vivadent AG, Schaan, Liechtenstein, Germany.	HfO_2_, Al_2_O_3_, Y_2_O_3_ and other oxides	Z010V8
	ZirLiner liquid		Mixture of butanediol, water and chloride	Z01JF0
Nanofluorapatite veneering ceramic	IPS e.max Ceram.	Ivoclar Vivadent AG, Schaan, Liechtenstein, Germany.	SiO_2_ > 60% wt. Additional contents: Al_2_O_3_, ZnO_2_, Na_2_O, K_2_O, ZrO_2_, CaO, P_2_O_5_, fluoride, and pigments	Y43417

### Disc preparation

Zirconia discs were cut using a microtome cutting machine (BUEHLER IsoMet®4000, USA), where an automatic linear blade was used with a speed 2350 rpm and feed rate 13.2 mm/min. The zirconia discs were sintered at 1500 °C as instructed by the manufacturer in a programmable furnace (MIHM-VOGT GMBH sintering furnace, Germany) and cleaned in an ultrasonic bath (VITASONIC II; Germany) filled with ethanol (99.5%) for 5 min.

Group I discs were air-particle abraded using 110 µ Al_2_O_3_ particles (Cobra Renfert-110 µ; GmbH Company, Germany) at 8 bar pressure.

**Fig. 1:**
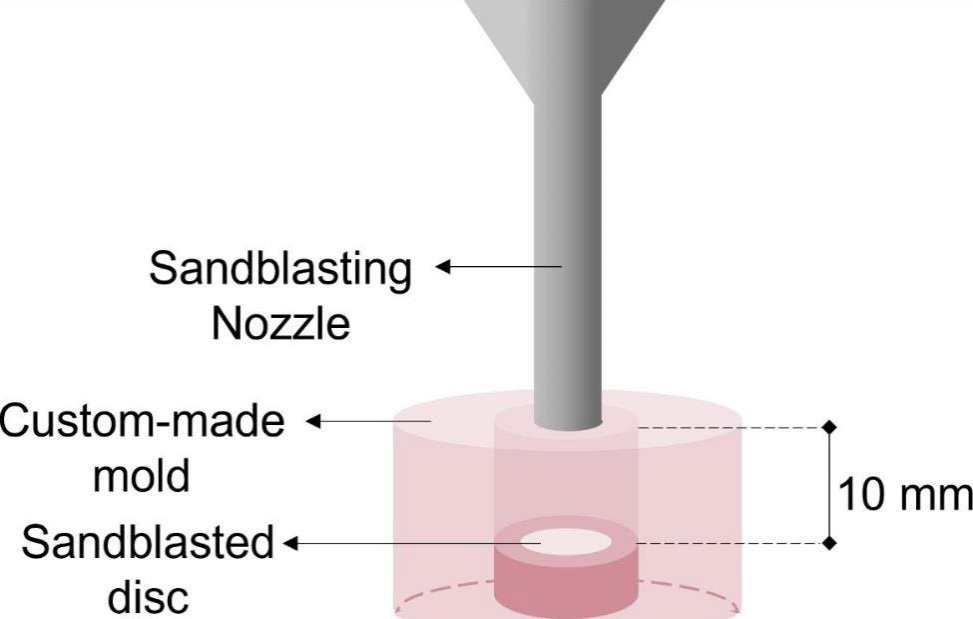
Schematic diagram of the sandblasting process

The working surface of each disc was abraded by placing the nozzle perpendicular to the disc surface, at a distance of 10 mm between the nozzle and the surface of the disc for 10 s. The distance was standardized by placing each disc in a custom-made cylindrical acrylic mold, at a depth of 10 mm. The sandblasting nozzle was held perpendicular to the disc with its tip flush with the opening in the cylinder as shown in Fig. 1.

After that, the zirconia surfaces were cleaned using 96% ethanol in an ultrasonic bath for 10 min to remove any loose particles caused by air-abrasion.

For group II, the discs were coated with bioglass coating (Bioglass nanoparticles; Nanostreams Egypt: NS0001). Each zirconia disc was coated using a brush to produce a uniform bioglass-coated layer, then allowed to dry for 2 h and fired at 1150 °C for 16 min in a furnace (Programat® P310; Liechtenstein, Germany).

group III, discs were coated with a liner (IPS e.max Ceram ZirLiner; Ivoclar Vivadent, Schaan, Liechtenstein, Germany) following to the manufacturer’s instructions. The powder and liquid were mixed and applied to form a uniform thickness on the entire framework, then fired at 960 °C in the furnace [24].

For group IV, discs were prepared using the wash firing (sprinkle) technique. In this technique, glazing material (IPS e.max CAD Crytsall./Shades,Stains, and Glaze Paste; Ivoclar Vivadent AG, Schaan, Liechtenstein, Germany) was mixed with the respective IPS liquids to the desired consistency and applied to form a covering layer on the entire framework. After that, the nanofluorapatite veneering ceramic (IPS e.max Ceram; Ivoclar Vivadent AG; Schaan, Liechtenstein, Germany) dentin powder was sprinkled on the restoration using a dry brush. The wash firing was performed on a honey-comb firing tray and fired at 750 °C in the furnace [17].

### Application of the veneering ceramic

For ceramic veneering, a teflon mold was constructed to adjust the dimensions of the veneering material (4 mm in diameter and 3 mm in height). Each zirconia disc was seated in the mold, then the nanofluorapatite veneering ceramic (IPS e.max Ceram; Ivoclar Vivadent AG; Schaan, Liechtenstein, Germany) was applied on the treated core surface till the desired height then fired in the furnace at 750 °C [17]. The discs were washed using an air–water spray then stored in distilled water at 37 °C for 24 h before shear bond strength testing.

### Microscopic and surface analysis

An additional zirconia disc from each surface treatment group prior to ceramic veneering was fabricated for scanning electron microscope (SEM) (Jeol JSM-IT200; Jeol Ltd, Tokyo, Japan) analysis. Each disc was coated with gold sputter coating in a machine before SEM examination. After gold coating, images were captured at magnifications of (500x and 1000x) with an accelerating voltage of 20 KV to examine surface morphology of each disc.

### SBS measurement

A universal testing machine (5ST; Tinius Olsen, England) was used for SBS testing to debond the zirconia discs from the veneering ceramics in accordance with ISO 17095:2013 [25] at a crosshead speed of 0.5 mm/min and with a 5-KN load cell. The constructed disc was secured in the testing device’s lower plate shape. A blade with a chisel-like form applied shear stress to the bonded area. Utilizing the machine’s compression mode, load could be applied to the bonded surface between the zirconia surface and the ceramic veneering interface.

The load in kilograms, at which the eroding ceramics detached from the zirconia surface, was displayed on a digital monitor. The SBS was calculated as fracture load (kg) divided by disc surface area (cm^2^), where area of the disc equals πr^2^. To calculate the SBS in megapascals (MPa), the resultant values were multiplied by 0.09807.

### Fracture analysis

**Fig. 2:**
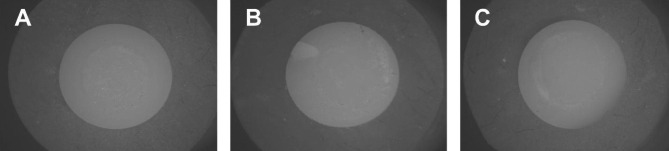
Stereomicroscopic image of the zirconia fractured sample demonstrating: A, an adhesive failure mood; B, a cohesive failure mode; C, a mixed failure mode

To determine the type of failure, the surfaces of the broken discs were examined under a stereomicroscope (SZ1145TR Olympus; Japan 1990) by using a software (Toup view,version 3.7). When there was no failure between the zirconia and veneering ceramic, the failure modes were observed as “adhesive”. “Cohesive” failures referred to those that occurred within the veneering ceramic, and “mixed” failures some ceramic was present, but zirconia was also exposed. Stereomicroscopic images of the zirconia fractured samples demonstrating each failure mode is shown in Fig. 2. The prevalence of each type of failure in each group and the statistical analysis is shown in Table 2.

**Table 2:** Comparison between the four studied groups according to mode of failure

Mode of failure	I (n = 13)		II (n = 13)		III (n = 13)		IV (n = 13)		χ^2^	^MC^ *P*
	No.	%	No.	%	No.	%	No.	%		
Adhesive	5	38.5	4	30.8	4	30.8	4	30.8	4.144	0.695
Cohesive	0	0.0	0	0.0	0	0.0	2	15.4		
Mixed	8	61.5	9	69.2	9	69.2	7	53.8		

χ^2^: Chi square test MC: Monte Carlo

*P* : *P* value for comparing between the four studied groups

### Statistical analysis

Normality was checked using descriptive statistics, plots and normality tests. Force and SBS showed normal distribution. Mean, standard deviation (SD), median, interquartile range (IQR) and range were calculated. Comparison between the four study groups was done using One-Way ANOVA followed by multiple pairwise comparisons using Bonferroni adjusted significance level. Significance was set at *P* value ≤ 0.05. Data were analysed using a statistical software (IBM SPSS v23.0 for Windows; IBM Corp).

## Results

**Fig. 3:**
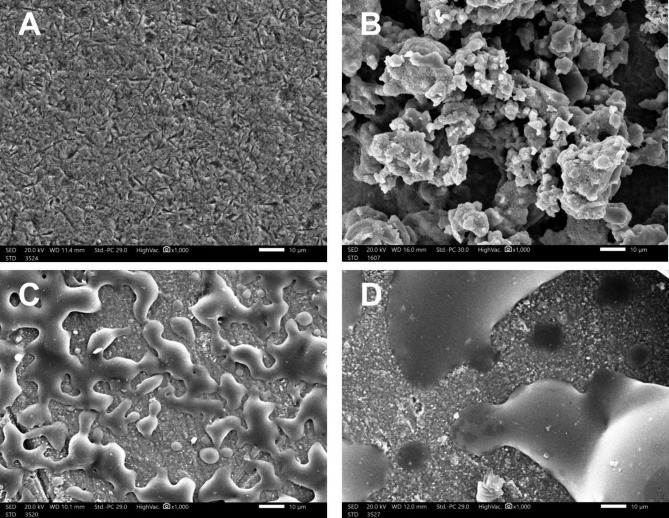
SEM images of zirconia surfaces before veneering application. **a** group I showing Y-TZP crystal structures magnifcation×1000. **b** group II showing clusters magnifcation×1000. **C** group III ZirL coating covering Y-TZP magnifcation×1000. **d** group IV sprinkles covering Y-TZP magnifcation×1000

SEM analysis of the treated discs showed some variations in surface topography among the different groups. In group I, zirconia crystal structures were observed in the air-abraded surface with a uniform distribution of pores and pits due to the effect of airborne particle abrasion. In group II, bioglass coated surfaces showed the formation of glass clusters in mesh-like pattern. In group III, small areas uncovered by the liner were observed with a uniform distribution. Group IV showed wash firing resulting in large areas uncovered by sprinkle particles Fig. 3.

Quantitative data were described using range (minimum and maximum), mean, SD and median. The mean SBS in MPa are presented in Table 3, showing that the highest mean bond strength was recorded in group III (17.98 ± 2.51 MPa), followed by group II (15.10 ± 4.53 MPa), then group I (14.65 ± 2.97 MPa) The lowest mean bond strength was recorded in group IV (13.28 ± 3.55 MPa).

**Table 3:** Comparison of shear bond strength between the four study groups

	I	II	III	IV
**Mean (SD)**	14.65 (2.97)	15.10 (4.53)	17.98 (2.51)	13.28 (3.55)
**Median (IQR)**	14.68 (3.03)	14.03 (6.11)	18.48 (2.98)	12.73 (5.66)
**Min – max**	8.95–20.16	9.17–24.55	12.35–21.04	7.38–19.60
**One-way ANOVA** ***P*** **value**	0.03*			
**Bonferroni adjusted post-hoc tests** ***P*** **value**	I vs. II: 1.00 I vs. IV: 1.00 I vs. III: 0.22 II vs. III: 0.34 II vs. IV: 1.00 III vs. IV: 0.02*			

*statistically significant at *P* value < 0.05

Bonferroni adjusted post-hoc tests revealed that there was no statistically significant difference in the bond strength values between any of the groups except between group III ( liner coating) and group IV (wash firing) as shown in Table 3.

## Discussion

The robust bonding between the ceramic veneer and zirconia core is essential for the predictable success of zirconia-based ceramic restorations. Mechanical laboratory studies are frequently the foundation for clinical advice on materials, processes, or methodologies. The shear bond test, which was also used to assess the bond strength between core and veneer in various ceramic systems, is one of the most used methods for determining bond strength. SBS test is used in this study because of ease of preparation of the samples and simple test protocol.

The surface finish of the core, which can affect mechanical retention, the emergence of flaws and structural defects at the core-veneer interface, the wetting properties, the volumetric shrinkage of the veneer, and the residual stresses caused by a mismatch in the CTE are just a few of the many factors that can affect the strength of the bond between the core and veneer. To produce a good interfacial bond strength, the core and veneering ceramic’s coefficients of thermal expansion must nearly match.

Zirconia core material underwent several surface treatments in this investigation to improve its adhesion to ceramic veneering. The null hypothesis, which claimed that there would be no significant difference in SBS between veneering ceramics and zirconium cores after different interfacial surface treatments, was rejected in light of this study’s findings.

A previous study by Al-Dohan et al. reported SBS in range of 22–31 MPa for commercially available core-veneer ceramic systems [26]. Another study reported SBS values in the range of 20-35 MPa [27]. These reported SBS ranges were slightly higher than the values obtained in our study as cylinders of smaller diameter of the veneering ceramic were used and. Consequently, the shear forces were divided by a smaller surface area leading to higher values of SBS in megapascals. On the contrary and in accordance with our study, Rivera et al. reported a lower SBS range of 11–15 MPa [28].

The highest shear bond strength was observed in group III ( liner coating group). Zirconia and ceramic are bonded together through a variety of mechanisms, including chemical and mechanical interlocking.The HFO_2_, Al_2_O_3_, Y_2_O_3_ and other oxides in the liner coating coating may enable for greater chemical interaction between the zirconia and the ceramic [5, 7] which may have increased the interfacial binding strength between the materials.

In accordance with results of the current study, López Mollá et al. evaluated the SBS of zirconia with a ZirLiner coating and a fluoroapatite-pressed ceramic and found a strength of 12.70 MPa, which is comparable to the findings of the present work (17.98 ± 2.51 MPa) [29]. Contrarily, other studies claim that a liner may have detrimental impacts on binding strength [8, 30, 31].

Group II (bioglass coating group) had lower shear strength values than the group III (liner coating group). This finding may be supported by a finding of a previous study by Rivera et al., which concluded that glass coatings enhanced bond strength between zirconia and fluorapatite veneering ceramic, improving chemical and mechanical interlocking [28].

Group I showed SBS even lower than those of groups II and III, yet it is worth noting that there was no significant difference between the SBS values of these 3 groups. In a previous study by Bagheri et al., novel phases such Al_2_SiO_5_ and zirconium aluminium oxide were produced using the sol-gel dipping coating approach using a silica and aluminosilicate sol-gel [32]. This suggests a chemical link between the veneering materials and the Y-TZP substrates, which strengthens the binding. Several other studies suggested that increasing the surface roughness of zirconia by using air abrasion increased ceramic bond strength by promoting the mechanical interlocking of the materials [33–35].

The lowest SBS values were shown in group IV. Findings of the present study concur with those of a previous where samples that fractured cohesively within a veneer had lower SBS values than samples that fractured cohesively within the core or had a mixed cohesive/adhesive fracture pattern [36]. In the current study, analysis of the failure mode revealed that group IV displayed a cohesive mode of failure within the weak veneering ceramic.

It is worth noting that, in the current study, the failure mode of the majority of the discs showed mixed cohesive/adhesive failure at a high percentage, significantly presented in groups II and III (69.2%). That is with the exception of group IV, which displayed a cohesive mode of failure within the weak veneering ceramic (15.4%) .Adhesive failure occurred at a low percentage (30.8% in groups II,III,IV and 38.5% in group I).

According to the results of the current study, on comparing between groups there was no statistically significant difference of the bond strength values between them except between groups III and IV. These investigations showed that various surface treatments improve the bonding between the zirconia core and veneering ceramic.

Because the test findings are influenced by the disc design, the type of mechanical testing, and the various materials utilised, it is difficult to compare different research. To the authors’ best knowledge, no previous studies examined and compared the effect of these surface treatments, specifically bioglass coating and wash firing (sprinkle technique), on SBS between zirconia and ceramic veneering using the same standardised mechanical testing methods.

Among of the limitations of the current study is that the disc design does not represent the clinical configuration of a zirconia ceramic restoration. Nevertheless, the design used in the study allowed for the SBS testing procedure under standardized conditions. Another limitation is that the in vitro nature of the study does not reflect the conditions of the oral cavity. Moreover, the bond strength was only evaluated using the shear bond strength test, while the mouth exposes restorations to other forces. Hence, additional studies mimicking the conditions of the oral cavity should be conducted with the aim of improving the bond strength between the zirconium core and ceramic veneering.

## Conclusions

1. Zirconia-veneer shear bond strength was significantly affected by different interfacial surface treatments.
2. The highest shear bond strength was found in the liner coating group, followed by the bioglass coating and air-borne particle abrasion groups. There was no significant difference between these 3 groups.
3. The lowest shear bond strength was shown in the wash firing group, significantly lower than the liner coating group.

## Data Availability

The datasets generated and analysed during the current study are available from the corresponding author on reasonable request.

## Abbreviations

Al_2_O_3_: Aluminium oxide
CTE: Coefficients of thermal expansion
IQR: Inter-quartile range
Kg: Kilogrammes
MPa: Megapascal
SEM: Scanning electron microscope
SBS: Shear bond strength
SD: Standard deviation
